# Should Leeds Have a Children's Hospital?

**Published:** 1913-07-26

**Authors:** 


### SHOULD LEEDS HAVE A CHILDREN'S HOSPITAL?

The Lord Mayor of Leeds not long ago suggested that the city is in need of a children's hospital, and the matter is attracting plenty of attention and discussion in the locality. Mr. Charles Lupton, chairman of the Leeds General Infirmary, and one of the ablest of present-day leaders in the institutional world, spoke last week against the proposal at a large meeting of the Leeds Luncheon Club. His main argument can be briefly summarised thus: It is desirable that any hospital should have the benefit of a chemical, bacteriological, and pathological laboratory; that a new hospital cannot be thoroughly equipped with these adjuncts without great expense; that such provision exists and is carried on with the utmost efficiency at the Leeds Infirmary; and that it is sounder business and better for the children that the work at the Infirmary should be supported and extended rather than that superfluous energy should be diverted towards a separate institution.

In this matter some people may hold that Mr. Lupton is biassed, but a close examination of his speech does not favour such a view. Mr. Lupton admits fully that Leeds needs more hospital accommodation for children; he even asserts that in the summer there is often very serious overcrowding in children's wards of the Infirmary, and his figures are proof positive that this is the case. Having granted this much, he proceeds to deny that the best remedy is the promotion of a separate children's hospital; and beyond question it would be far the cheaper plan to extend the Infirmary accommodation by the necessary number of beds. It will be argued that the tendency of the day in large and progressive cities is towards the multiplication of special hospitals, including children's hospitals, and that this on the whole makes for greater efficiency. We doubt whether there is a great deal in this argument as applied to so large an institution as the Leeds General Infirmary. The children's wards there now contain seventy-six cots; with twenty or thirty added, there would be few larger clinics in England in this specialty. A separate out-patient department for the children would complete this hospital within a hospital and justify Mr. Lupton's position. At the same time there is undoubtedly much to be said on the other side of the question, and the analogy of London certainly supports the Lord Mayor's solution of the problem of providing the best care for the sick children of Leeds. As a matter of fact, a great many of the medical and surgical staff of the London children's hospitals—indeed, the great majority—hold also one or more appointments at general hospitals. It cannot, therefore, be argued that medical men must or should stick to this particular specialty in the same way that they stick to ophthalmology, gynæcology, and similar specialised departments of medicine and surgery. And if a children's hospital were to be started in Leeds we should be fairly safe in prophesying that most of the honorary staff would be drawn from the Infirmary staff. On the whole, therefore, we think that Mr. Lupton is in the right in this matter, though there is admittedly room for difference of opinion.

